# Direct Modification of Extracellular Vesicles and Its Applications for Cancer Therapy: A Mini-Review

**DOI:** 10.3389/fchem.2022.910341

**Published:** 2022-05-11

**Authors:** Wenbin Nan, Chao Zhang, Hao Wang, Hongli Chen, Shenglu Ji

**Affiliations:** ^1^ College of Life Science and Technology, Nano Biomedical Materials Research Center, Xinxiang Medical University, Xinxiang, China; ^2^ The Third Hospital of Xinxiang Medical University, Xinxiang, China

**Keywords:** extracellular vesicles, cancer therapy, drug delivery, modification, nanomedicine

## Abstract

Extracellular vesicles (EVs) are a class of lipid membrane-bound vesicles released by various cells and mediate cell-to-cell communication. By reason of their high physiochemical stability and biocompatibility, EVs are considered as novel drug delivery system. An increasing number of studies have indicated that EVs can be modified to enhance their loading efficiency, targeting ability and therapeutic capabilities for cancer therapy. Compared with the tedious process of gene engineering approaches, direct modification of EVs is easier, faster and versatile. This mini review will summarize the prevailing approaches for direct modification of EVs. Additionally, the potential applications of modified EVs in cancer therapy are also discussed, which will help readers gain a better understanding of the technologies and applications in this field.

## Introduction

Aberrant proliferation and aggressive metastatic capacity are the hallmarks of malignant tumors (Hanahan and Weinberg, 2011). The conventional oncological treatment options such as surgery, radiation, and chemotherapy are associated with several disadvantages, including poor targeting and severe side effects (DeVita and Chu, 2008). On the other hand, treating metastatic cancers with a high dose of drugs may result in drug resistance of cancer cells (Aleksakhina et al., 2019). Therefore, novel therapeutic approaches need to be developed to improve the efficiency, specificity and safety of cancer therapy.

Extracellular Vesicles (EVs) are nano membrane vesicles released from various cells. EVs can carry complex cargos such as proteins, lipids and nucleic acids, and communicate directly with recipient cells (Maas et al., 2017). In addition, EVs have some special advantages, including escaping from clearance by host immune system and passing through physiological barriers (Colao et al., 2018; Liu et al., 2019), which makes them suitable to be used as potential therapeutic agents and drug delivery vehicles (Liao et al., 2019). However, limitations remain in the use of natural EVs for cancer therapy. For example, the low targeting capacity of EVs might seriously affect the therapeutic effect, and a heterogeneous range of molecules contained in EVs brings safety concerns (Jabalee et al., 2018). At present, the EVs used in the studies for cancer treatment mainly derived from tumor cells, stem cells or immune cells. EVs derived from tumor cells exhibit relatively good targeting but with a risk (Taghikhani et al., 2020), whereas EVs derived from stem cells or immune cells have a good therapeutic effect but lack of targeting (Herrmann et al., 2021).

To circumvent these problems, modified EVs have recently emerged as a new alternative strategy for cancer therapy (Vader et al., 2016). Accumulating evidence suggests that the modification of cargo loading or membrane components of EVs enhances their loading efficiency, targeting ability and therapeutic capabilities (Yong et al., 2020). In general, the approaches of modifying the EVs can be classified into direct modification (directly remold EVs) and indirect modification (engineer the parent cells). Compared with the tedious process of gene engineering approaches, direct modification of EVs is more simple, rapid and versatile (Nie et al., 2021). In this review, we focus on the prevailing approaches for direct modification of EVs, including cargo loading and membrane modifying of the EVs. Additionally, the latest reported progress in the applications of modified EVs for cancer therapy are summarized and discussed.

## Direct Modification of EVs

### Cargo Loading Strategy for EV Modification

The approach to load exogenous cargo into EVs can be divided into passive and active loading methods. The passive loading method refers to therapeutic drugs directly incubated with EVs (Zhu et al., 2019) or donor cells (Guo et al., 2019; Zheng et al., 2020). Generally, the hydrophobic molecules are prone to interact with lipids exposed on EV membrane surface, making passive co-incubation the best approach for hydrophobic drugs with poor solubility. Although these methods are straightforward and do not damage the structure of EVs, the loading efficiency depends on the drug properties, incubation periods and other details. For example, curcumin and cucurbitacin-I were shown to rapidly diffuse into EVs when they were incubated at 22°C for 5 min, and the EV encapsulation could penetrate through the blood-brain barrier to exert anti-tumor effects in the glioblastoma model (Sun et al., 2010; Zhuang et al., 2011).

Active loading method refers to therapeutic drugs crossed through the EV membrane by electroporation (Zou et al., 2019), sonication (Zhupanyn et al., 2020), freeze and thaw cycles (Haney et al., 2015), extrusion (Bose et al., 2018), and so on. Based on these methods, a variety of therapeutic drugs have been loaded into EVs for the treatment of refractory tumors. The drug-loaded EVs promote the accumulation of drugs in cancer cells, enhancing blood circulation time, and consequently improving their treatment outcomes. Kim et al. compared these common methods, the results suggested that all the active loading methods attained higher loading efficiencies than the passive loading method, especially sonication and extrusion methods (Kim et al., 2016). However, these methods also have some limitations. For instance, during the electroporation and thaw cycle process, the media that contains phosphate-buffered pulse or sucrose could cause EV aggregation (Yan et al., 2020). In addition, EV membrane properties may be damaged due to the extrusion method (Fuhrmann et al., 2015). Thus, the most appropriate loading method for a target molecule depends on its physicochemical properties. For example, the passive loading method is typically suitable for small molecule and hydrophobic drugs because it can cross the hydrophobic membrane of EVs (Haney et al., 2015). For small RNA cargos, electroporation is the best loading approach because of its higher loading efficiency (Lamichhane et al., 2015), while the methods such as extrusion and sonication are more suitable for larger proteins and hydrophilic molecules (Yuan et al., 2017). The cargo loading strategies for EV modification are given in Table 1.

**TABLE 1:** Cargo loading strategies for EV modification.

Strategies	Methods	Advantages	Disadvantages	Examples	References
Passive loading	Co-incubation with EVs	Straightforward; No damage to the structure of EVs	Poor specificity; Low loading efficiency	Hydrophobic molecules such as Curcumin and Cucurbitacin-I	Sun et al. (2010); Zhuang et al. (2011)
	Co-incubation with donor cells	Straightforward; No damage to the structure of EVs	Poor specificity; Low loading efficiency	Hydrophobic molecules such as DOX and PTX	Guo et al. (2019); Zheng et al. (2020)
Active loading	Electroporation	Simple and quick; High loading efficiency	EV aggregation	Small RNA such as siRNA, shRNA	Zou et al. (2019); Lamichhane et al. (2015)
	Sonication	Relatively high loading efficiency	EV aggregation	Protein such as catalase; Hydrophobic molecules such as DOX	Zhupanyn et al. (2020); Lee et al. (2019)
	Freeze and thaw cycles	Simple and quick	EV aggregation; low loading efficiency	Protein such as catalase	Haney et al. (2015)
	Extrusion	Relatively high loading efficiency	Damage the membrane properties	Protein such as catalase; Hydrophobic molecule such as porphyrins	Bose et al. (2018); Fuhrmann et al. (2015)

### Modification of the EV Membrane

The approaches of direct modification of EV membrane are broadly divided into covalent and non-covalent modification. The covalent modification enables functional groups rapidly form covalent bonds with EVs. For example, sulfhydryl is widely presented on the EVs surface, therefore, it can be employed as the binding site for EVs labeling via the michael addition reaction between maleimide and sulfhydryl. Fan et al. utilized this method to anchor quantum dots (QDs) onto the surface of exosomes. They found that the QDs-labeled exosome complex can be swiftly engulfed by tumor cells, and the tumor cells were lighted up by the fluorescence of this complex (Fan et al., 2019). Click chemistry is a copper-catalyzed azide alkyne cyclo-addition reaction under physiological conditions (Kolb et al., 2001), and is also commonly used to enable bioactive molecules to form chemical bonds with EVs. Jia et al. conjugated the membrane of EV with neuropilin-1 targeted peptide (RGERPPR, RGE) by click chemistry to obtain glioma targeting EVs (Jia et al., 2018). However, these modification approaches might change the physicochemical properties of the EV membrane. The long-term biocompatibility, stability and safety of modified EVs still need in-depth research.

EVs can also be non-covalently modified based on their natural features. The membrane of EVs mainly consists of amphiphilic substances such as phospholipids, cholesterol and glycolipids, therefore, EVs allow hydrophobic compounds to integrate into their membrane by hydrophobic interaction. For instance, Cheng et al. integrated nuclear localization signal peptides on EVs surface by shaking the peptide and EVs in an ice bath for 4 h. The modified EVs exhibit a great enhanced therapeutic effect on the inhibition of tumor growth (Cheng et al., 2019). The EVs surface is negatively charged, as a result, positively charged components can bind to the surfaces of EVs via electrostatic interaction. Zhan et al. bind cationic endosomolytic peptides L17E to the exosome membrane through electrostatic interaction. The modified exosomes exhibit an enhanced tumor accumulation, thereby efficiently delivering encapsulated cargos to tumor cells (Zhan et al., 2020). Besides, ligand-coupled molecules can specifically bind to receptors expressed on the EV surfaces. For example, transferrin receptors (TfR) are enriched at the membranes of EV derived from reticulocyte. Yang et al. synthesized a pH-responsive superparmagnetic nanoparticles cluster (SMNC), and bind to blood TfR-positive exosomes by precisely labeled with transferrin receptor (Yang L. et al., 2019).

## Application of Modified EVs in Cancer Therapy

**FIGURE 1:**
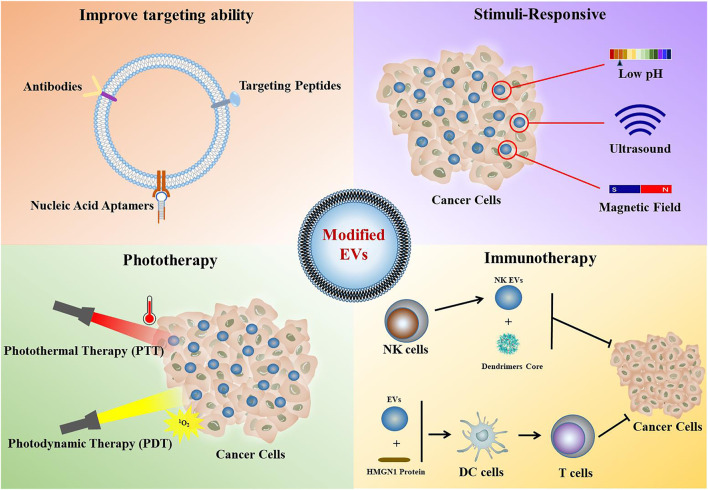
Application of modified EVs in cancer therapy.

Based on these methods of modifying EVs, many researchers have developed new methodologies to modify and design EVs to improve their targeting ability, loading efficiency and therapeutic efficacy (Figure 1). In this subsection, we summarized the latest reported progress in the applications of modified EVs for cancer therapy.

### Enhancement of Cell Targeting Specificity of EVs

Generally, EVs have natural targeting properties, this is mainly due to that EVs contain and transfer multiple bioactive molecules from their derived cell lineage. Wang et al. (2021) reported that neutrophil-derived EVs possess appealing blood-brain barrier penetration capability, and can aid delivery of the doxorubicin (DOX) facilely enter into brain and target to glioma via clathrin endocytosis (Wang et al., 2021). However, the intrinsic targeting capacity of most natural EVs is still unsatisfactory. The most direct strategy for improving targeting is to modify the antibodies onto the surface of the EV membrane. Nevertheless, antibodies are rarely used for targeting directly, because of their large size, complex structure and high risk of generating an immune response (Ahmad et al., 2012). The relevant research has mainly focused on simpler fragments of antibodies such as single domain antibodies (sdAbs) (Pham et al., 2021) or single chain variable fragments (scFvs) (Longatti et al., 2018; Wang et al., 2018). Results showed that these approaches could effectively improve the tumor targeting of EVs. Compared with antibodies, targeting peptides provide several advantages including small size and lower immunogenicity (Hung and Leonard, 2015). Some peptides have been utilized to target tumor associated receptors. For example, RGD peptide can be used for glioblastoma targeted therapy via target integrin receptors (Zhu et al., 2019), and the mesenchymal-epithelial transition factor (c-Met) binding peptides can be used for triple negative breast cancer targeted therapy via target c-Met (Li et al., 2020). Besides, nucleic acid aptamers are small synthetic single stranded DNA or RNA molecules which are also capable of binding selectively to target molecules (Zhang et al., 2021). Similar to targeting peptides, nucleic acid aptamers also possess advantages such as smaller size, lower immunogenicity and simple chemical modification. For example, Zou et al. modified the EVs with diacyllipid-sgc8 aptamer which can specifically bind protein tyrosine kinase 7 (PTK7) through hydrophobic interaction, and the modified EVs can efficiently deliver molecular drugs/fluorophores to target cancer cells (Zou et al., 2019). The strategies for enhancing the targeting of EVs are given in Table 2.

**TABLE 2:** Strategies for enhancing targeting of EVs.

Strategies	Advantages	Disadvantages	Examples	Cancer Type	References
Antibody	Strong specificity	Large size; complex structure; high risk of generating an immune response	Anti-A33 antibody	Colorectal cancer	Li et al. (2018)
Antibody derivative	Simpler and more compact; Relatively low immunogenicity	The preparation process is complex and costly; There is still a risk of immunogenicity	Anti-EGFR sdAbs; Anti-HER2 scFv	Lung cancer; Breast cancer	Pham et al. (2021); Longatti et al. (2018)
Targeting peptides	Small size; easily synthesized and manipulated	Poor stability; susceptible to degradation or hydrolysis	RGD peptide; cMBP peptide	Glioblastoma; Triple negative breast cancer	Zhu et al. (2019); Li et al. (2020)
Nucleic acid aptamers	Small size; greater stability; lower immunogenicity and toxicity; simple chemical modification	The long-term biocompatibility, stability, and safety remains to be clarified	EGFR RNA aptamer; Sgc8 DNA aptamer	Breast cancer; T-cell leukaemia	Pi et al. (2018); Zou et al. (2019)

### Construction of Stimuli-Responsive EVs

Natural EVs cannot respond to exogenous stimulations, which limit their application in drug controlled release. Multiple studies have been carried out to improve the stimuli-responsive ability of EV-based nanoparticles.

Low extracellular pH is considered a key feature of tumor microenvironment (Kato et al., 2013). Modified EVs with pH-responsive materials altered their physicochemical characteristics which makes EVs respond to acidic pH of the tumor microenvironment, and further leads to sustained drug release at the tumor site. The intercalated motif (i-motif) is a pH-responsive DNA strand. Jun et al. constructed a pH-responsive delivery system by chemical modification of exosomes with biotin and ds-i-motif-bio conjugation via streptavidin on the surface of the exosomes. This system efficiently released DOX in an acidic pH responsive manner and had intact bioactivity for anti-proliferation to MCF-7 cells (Jun et al., 2018). Lee et al. designed a functional EV originated from RAW 264.7 cells by attaching a pH-responsive 3-(diethylamino) propylamine (DEAP) via sonication. The DEAP is protonated below pH 7.0, therefore, the functional EV would release drugs when its membrane disruption in response to the acidic pH of the tumor microenvironment (Lee et al., 2018). This work was further extended to target dendritic cells for anticancer vaccination, and the nanoparticle showed pH-dependent physicochemical characteristics which is consistent with the expectations (Lee et al., 2019).

Recently, there are some findings about ultrasound responsive EVs which warrant further attention. Liu et al. designed a functionalized smart nanoparticle in conjunction with an extracorporeal ultrasound device for tumor specific sonodynamic therapy. This nanoparticle was prepared by utilizing exosomes loaded with sinoporphyrin sodium (DVDMS) via a very mild incubation. Results indicated that this ultrasound-responsive natural exosome-based delivery system can non-invasively enhance homogenous tumor targeting and sonodynamic therapy toxicity (Liu et al., 2019). Sun et al. revealed that ultrasound microbubbles together with ultrasound-targeted microbubble destruction (UTMD) significantly increase the infiltration and endocytosis of EVs in these reluctant tissues such as heart and adipose tissue (Sun et al., 2019; Sun et al., 2020). These techniques may have potential applications for anti-cancer EV-based drug delivery.

Magnetic targeting is an important approach of passive targeting for tumor therapy. EVs functionalized by minute magnets could be enriched at the tumor site with the help of external magnetic fields (Xiong et al., 2021). One study from Qi et al. utilized superparamagnetic nanoparticles anchored onto EVs through Tf-Tf receptor interaction, and those modified EVs exhibited superparamagnetic behavior at room temperature. Furthermore, DOX was loaded into EVs, and these EV-based vehicles show excellent tumor targeting ability and cancer inhibition effect (Qi et al., 2016).

### Modified EVs for Phototherapy

As non-invasive methods of phototherapy, photothermal therapy (PTT) and photodynamic therapy (PDT) have high clinical value in the cancer therapy. A number of studies have loaded photothermal materials or photosensitizers into EVs so that these functionalized EVs can be used for PTT or PDT of tumors. For PTT, the photothermal conversion material could convert light energy into cytotoxic heat energy to kill cells (Wu et al., 2015). Cao et al. synthesized small fluorescent quantum dots (QDs) as the photothermal conversion material and modified with cell nucleus-target TAT (transactivator of transcription) peptides, then, packaged into RGD-EVs via electroporation to construct a versatile theranostic platform. This system mediated nucleus temperature increase under NIR-II region laser irradiation, leading to killing the breast cancer cells completely (Cao et al., 2019). Wang et al. have co-embedded Bi_2_Se_3_ and DOX into tumor cell derived microparticles by electroporation method, and obtained Bi_2_Se_3_/DOX@MPs via irradiation-induced budding. Bi_2_Se_3_/DOX@MPs exhibit remarkably dual-modal imaging capacity and synergistic antitumor efficacy by combining PTT with low-dose chemotherapy (Wang et al., 2020).

For PDT, the photosensitizer transfers energy from light to molecular oxygen to generate singlet oxygen, which is toxic to cancer cells (Yang R. et al., 2019). Pan et al. developed a novel nanovehicle by combining urinary EVs and Au-BSA@Ce6 nanocomposites via electroporation (Pan et al., 2020). The structures of nanovehicles collapsed under 633 nm laser irradiation, and a large number of nanoparticles were released to produce singlet oxygen in cancer cells that in turn result in suppression of tumor growth. Zhu et al. use electroporation method to prepare a hybrid nano-vesicle by loaded aggregation-induced emission (AIE) molecular onto tumor-derived EVs, this hybrid nano-vesicles could facilitate efficient tumor penetration and significantly enhance the PDT effect (Zhu et al., 2020). These findings indicate that modified EVs with rational design provide novel approaches to cancer therapy.

### Modified EVs for Immunotherapy

Tumor immunotherapy has gained increased attention in recent years. The modifications of EVs derived from immune cells such as natural killer cells (NKs), dendritic cells (DCs) have been used for tumor immunotherapy. Wang et al. report a novel strategy based on NK cell derived exosomes (NKEXOs) for tumor targeted therapy. The biomimetic core-shell nanoparticles (NNs) were self-assembled with a dendrimers core loading therapeutic miRNA and a hydrophilic NKEXOs shell, the resulting NN/NKEXO showed highly efficient targeting and therapeutic miRNA delivery to neuroblastoma cells, leading to inhibit tumor growth (Wang et al., 2019). High mobility group nucleosome binding protein 1 (HMGN1) can enhance the ability of DCs to activate T cells and improve vaccine efficiency (Yang et al., 2012). Zuo et al. modified tumor-derived EVs with the functional domain of HMGN1 via an anchor peptide, and DCs pulsed by these modified EVs show long-term anti-tumor immunity and tumor inhibition effect by enhancing memory T cell response (Zuo et al., 2020). The chimeric antigen receptor T (CAR-T) cell therapy is a new strategy in adoptive antitumor treatment. CAR-T therapy can induce rapid and durable clinical responses but associated with acute toxicities. Fu et al. report that EVs derived from CAR-T cells carry CAR on their surface, and express a high level of cytotoxic molecules to induce tumor cell death. More importantly, CAR EVs do not express Programmed cell Death protein 1 (PD1), and their antitumor effect cannot be weakened by recombinant PD-L1 treatment, and that is why the administration of CAR EVs is relatively safe compared with CAR-T therapy (Fu et al., 2019). In summary, modified EVs have broad application prospects in tumor immunotherapy.

## Perspectives

Despite EVs of natural origin having advantages such as good biocompatibility, low toxic side effects and good blood-brain barrier penetration, many questions remain to be answered, including insufficient loading efficiency and poor targeting. Approaches for direct modification of EVs brought new lights on resolving these problems. In recent years, an increasing number of studies have used new technologies to design and modify EVs to improve their loading efficiency and targeting ability, and these modified EVs indeed have shown exciting and encouraging results in both experimental and preclinical studies as anticancer drugs. However, the findings of some EV clinical trials did not live up to anticipated outcome, which suggest that most applications are still at an experimental stage. Because of the heterogeneity of the encapsulated and surface molecules, the use of different isolation, purification and characterization methodologies frequently results in confusion with regard to characteristics of EVs. In addition, in initial lack of standardized protocols for EV modification, resulting in contrasting results between different studies. Therefore, the standard for isolation, purification, characterization and modification of EVs need to be established. On the other hand, the functional molecules carried by EVs may bring potential biosafety problems, so that these EVs based therapeutic strategies require further preclinical research before successful clinical application. Overall, EVs based therapeutic strategies for cancer are still in their infancy, with the deepening of basic research on EVs and the development of biotechnology, the applications of modified EVs for cancer therapy are potentially broad.
